# Considerations for Measuring Activity-Dependence of Recruitment of Synaptic Vesicles to the Readily Releasable Pool

**DOI:** 10.3389/fnsyn.2019.00032

**Published:** 2019-11-20

**Authors:** John F. Wesseling

**Affiliations:** CSIC/Instituto de Neurociencias, Universidad Miguel Hernández, Alicante, Spain

**Keywords:** RRP, readily-releasable, residual, activity, recruitment, vesicle, pool

## Abstract

The connection strength of most chemical synapses changes dynamically during normal use as a function of the recent history of activity. The phenomenon is known as short-term synaptic plasticity or synaptic dynamics, and is thought to be involved in processing and filtering information as it is transmitted across the synaptic cleft. Multiple presynaptic mechanisms have been implicated, but large gaps remain in our understanding of how the mechanisms are modulated and how they interact. One important factor is the timing of recruitment of synaptic vesicles to a readily-releasable pool. A number of studies have concluded that activity and/or residual Ca^2+^ can accelerate the mechanism, but alternative explanations for some of the evidence have emerged. Here I review the methodology that we have developed for isolating the recruitment and the dependence on activity from other kinds of mechanisms that are activated concurrently.

## 1. Introduction

Presynaptic terminals typically contain hundreds of vesicles laden with neurotransmitter, but, at any given time, only a few per cent are docked at the plasma membrane and are ready to undergo exocytosis on demand. These *readily releasable* vesicles are often described as constituents of a *readily releasable pool* (RRP). High frequency trains of action potentials can deplete the RRP at a broad range of synapse types by driving exocytosis more quickly than replacement vesicles are recruited from reserve stores. The depletion is one of the mechanisms that causes short-term synaptic depression, although not the only one; e.g., inactivation of presynaptic voltage-gated Ca^2+^-channels and desensitization of postsynaptic neurotransmitter receptors play important additional roles at some types of synapses (Trussell et al., 1993; Otis et al., 1996; Chen et al., 2002; Nanou and Catterall, 2018).

The Ca^2+^ that enters presynaptic terminals during the high frequency trains is cleared slowly during subsequent periods of rest; this is termed *residual Ca*^2+^. A number of studies have concluded that the residual Ca^2+^ can accelerate how quickly replacement vesicles are recruited to the RRP (Kusano and Landau, 1975; Dittman and Regehr, 1998; Stevens and Wesseling, 1998; von Gersdorff et al., 1998; Wang and Kaczmarek, 1998; Gomis et al., 1999; Wang and Manis, 2008; Babai et al., 2010; Johnson et al., 2017). However, the techniques used to measure the timing of recruitment vary greatly between research groups, and the details matter because some techniques generate estimates that are 10-fold faster than others (discussed in Garcia-Perez and Wesseling, 2008).

Some of the discrepancies may be semantic. For example, RRPs are thought to contain separate slow-releasing and fast-releasing subdivisions; vesicles within slow-releasing subdivisions are sometimes termed *reluctantly releasable* (Wu and Borst, 1999; Moulder and Mennerick, 2005; Neher, 2017; see Taschenberger et al., 2016 for evidence for a third subdivision). Some models, termed *serial* models, include the premise that vesicles recruited to the fast-releasing subdivision are drawn from vesicles already within the slow-releasing subdivision (Wu and Borst, 1999; Sakaba and Neher, 2001; Hosoi et al., 2007; Lee et al., 2010; Miki et al., 2016). If so, some widely used techniques may measure the transfer of vesicles from the slow-releasing subdivision to the fast-releasing subdivision rather than from entirely outside the RRP to within any of the subdivisions.

Nevertheless, at least two alternative explanations have been proposed for some of the evidence used to conclude that residual Ca^2+^ accelerates recruitment from outside the RRP entirely to within any subdivision, which we term *recruitment to the RRP as a whole*. Here I first describe the underlying caveats and then our own strategy for measuring the timing and why we remain confident that residual Ca^2+^ truly does accelerate the underlying mechanism, at least at excitatory hippocampal synapses.

Note that the concept of recruitment to the RRP as a whole will be equivalent to recruitment to the slow-releasing subdivision if a serial model turns out to be correct. I use the more general terminology here, however, because we remain open to the possibility that slow- and fast-releasing subdivisions may instead operate wholly in parallel. Indeed, results from experiments on calyces of Held indicate that neurotransmitter in both slow- and fast-releasing subdivisions can be released directly with sub-millisecond timing; neurotransmitter in the slow-releasing subdivision is released slowly during trains of action potentials at least partly because the fraction of slow-releasing vesicles that undergo exocytosis directly after each action potential - termed the *probability of release per vesicle*- is low (see Figure 5-figure supplement 1 in Raja et al., 2019). The results do not rule out serial models where slow-releasing vesicles can be recruited to the fast-releasing subdivision in addition to undergoing exocytosis directly, but we believe that our conclusions are equally valid whether or not transfer between subdivisions can occur (Mahfooz et al., 2016).

## 2. The Caveats

**Figure 1:**
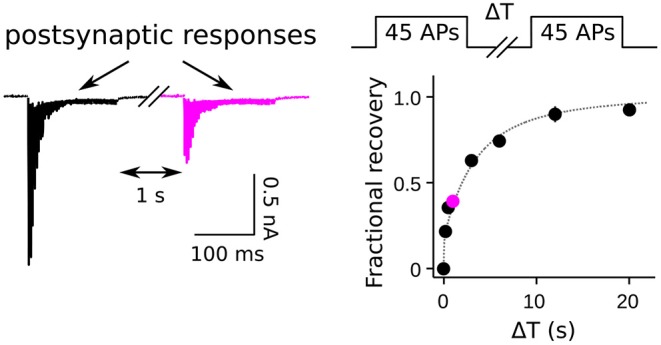
Measurement of RRP replenishment using pairs of trains of presynaptic action potentials. Left panel is an example of postsynaptic responses when the inter-train interval was 1 s at a calyx of Held synapse. Right panel is the time course of recovery extracted from trials where the length of the inter-train interval (ΔT) was varied. The magenta data point corresponds to the example at left. Adapted from Figure 8 of Mahfooz et al. (2016).

The caveats for measuring activity-dependence of recruitment to the RRP as a whole both pertain to experiments where the timing of recruitment is estimated from the time course of recovery after inducing short-term depression. In these cases, recovery is mainly measured from synaptic output generated by pairs of stimuli separated by interleaved rest intervals (Figure 1). The first stimulus induces the depression, and the second is used to assess the amount of recovery during the rest interval, typically by dividing the aggregate postsynaptic response generated by the second stimulus by the response generated by the first. The full recovery time course is then estimated from the individual trials as a function of the length of the rest interval; if significant recruitment occurs during stimulation, the offset can be estimated from interleaved trials where the rest interval is zero (Wesseling and Lo, 2002). A variety of stimuli have been used, including: single or multiple action potentials in trains; direct depolarization of presynaptic terminals via voltage clamp; indirect depolarization by applying hyperkalemic solution; and even osmotic shocks. All except osmotic shocks drive exocytosis by admitting Ca^2+^ into the presynaptic terminals. Evidence that the Ca^2+^ chelator EGTA lengthens the time course of recovery is interpreted as evidence that the recruitment mechanism is normally accelerated by the residual Ca^2+^ remaining after the first stimulus of each pair. However, the conclusion depends on the assumption that recovery from depression is equivalent to RRP replenishment, and this is not necessarily correct when the stimuli are not sufficient to fully exhaust all subdivisions of the RRP.

### 2.1. Enhanced Fusogenicity

**Figure 2:**
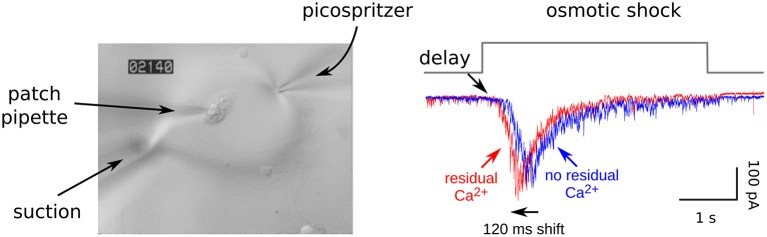
Residual Ca^2+^ accelerates release elicited by osmotic shocks. **Left:** Image of an autapse during a strong osmotic shock. Hypertonic solution was applied with a picospritzer for rapid onset combined with a suction pipette for equally rapid clearance, and was visible via DIC microscopy. **Right:** Postsynaptic responses during osmotic shocks. The leftwards shift after generating residual Ca^2+^ indicates that the Ca^2+^ enhances fusogenicity of readily releasable vesicles by lowering the energy barrier that prevents spontaneous exocytosis. In this experiment, residual Ca^2+^ was generated by firing a train of action potentials ending immediately before the recording begins, and caused 4-fold enhancement in the probability of release; RRP depletion was avoided because the extracellular Ca^2+^ was lowered to 0.25 mM. EGTA blocks the leftwards shift and enhancement of synaptic strength but does not alter the length of the delay in the absence of residual Ca^2+^ (not shown). See Figure 4 of Stevens and Wesseling (1999a) for a more extensive analysis and Figure 8 of Garcia-Perez and Wesseling (2008) for an analogous experiment when the readily releasable vesicles were new recruits.

The first caveat arose from the finding that residual Ca^2+^ activates a mechanism that transiently enhances the fusogenicity of readily releasable vesicles at hippocampal synapses (Stevens and Wesseling, 1999a; Garcia-Perez and Wesseling, 2008; e.g., see Figure 2). Enhancement of synaptic strength above baseline is typically masked during intense stimulation because of concurrent depletion of the RRP. However, the mechanism can enhance synaptic strength above baseline when conditions are manipulated to minimize the depletion, and is likely to be at least one of the causes of the classically defined element of short-term enhancement termed *Augmentation* (Magleby, 1979; Regehr, 2012). The role in information processing has not yet been resolved, but the mechanism does transiently sharpen the frequency filtering properties of synaptic transmission on a time scale of seconds.

The amount of enhancement of fusogenicity of individual readily releasable vesicles does not seem to be related to the extent of RRP depletion or subsequent replenishment, even when only recently recruited vesicles are present, suggesting that the mechanism operates independently from recruitment mechanisms (Garcia-Perez and Wesseling, 2008). Even extensive use that depletes the reserve stores and slows the bulk rate of recruitment by a factor of 3—termed *supply-rate depression* (Garcia-Perez et al., 2008)—does not dampen the enhancement for individual vesicles once they have entered the RRP (Garcia-Perez and Wesseling, 2008).

**Figure 3:**
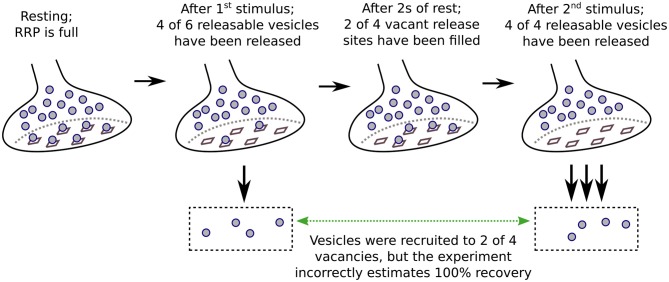
Enhanced fusogenicity can interfere with measurements of RRP replenishment.

Naturally, the impact of residual Ca^2+^ is largest when the concentration is high. As a consequence, the second stimuli of pairs that fail to completely exhaust the RRP drive exocytosis of a greater fraction of the readily releasable vesicles if applied after short rest intervals, when the RRP has only partially replenished, than after long rest intervals when replenishment is complete but fusogenicity is no longer enhanced because most of the residual Ca^2+^ has been cleared away (Figure 3). In this way, responses rebound from depression more quickly than RRP replenishment, and can even transiently overshoot baseline values (Garcia-Perez and Wesseling, 2008). EGTA would be expected to reduce the mismatch between recovery from depression and RRP replenishment by preventing the enhancement of fusogenicity. This would slow recovery even if the recruitment mechanism were not accelerated by Ca^2+^.

### 2.2. Fast Replenishment of EGTA-Sensitive Subdivisions

The mechanism that enhances fusogenicity either is not present or does not remain active long enough to influence recovery from depression after short trains of action potentials at calyces of Held (Mahfooz et al., 2016), and possibly other types of synapses. However, Ritzau-Jost et al. (2018) have identified a second caveat that could be equally problematic. In some cases, EGTA is thought to selectively block release of vesicles from the slow-releasing subdivision of the RRP, while leaving release from the fast-releasing subdivision intact, likely owing to details about how quickly the EGTA chelates free Ca^2+^ (Adler et al., 1991; Neher, 1998). When this occurs, treating with EGTA would change the measurement of recovery from a measurement of replenishment of the RRP as a whole to a measurement of replenishment of only the fast-releasing subdivision. This could be problematic because the fast-releasing subdivision is thought to be replenished more slowly than the slow-releasing subdivision at some types of synapses (Wu and Borst, 1999; Sakaba and Neher, 2001; Lee et al., 2013; Ritzau-Jost et al., 2018; but see Garcia-Perez and Wesseling, 2008; Mahfooz et al., 2016). Here again, EGTA could slow recovery without necessarily altering the underlying timing of recruitment of vesicles to the RRP.

## 3. Detecting Acceleration With Osmotic Shocks

**Figure 4:**
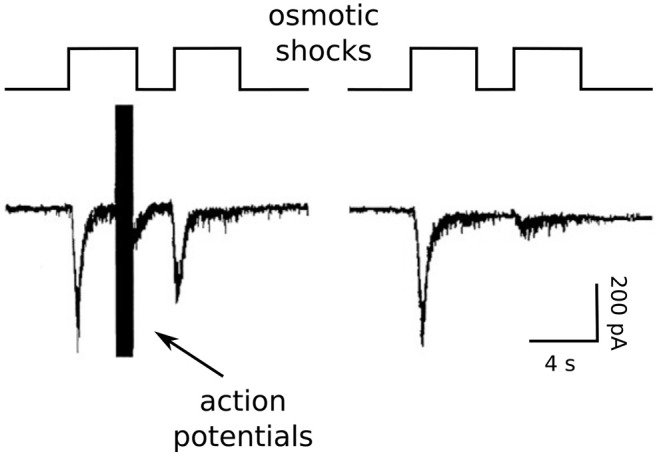
Osmotic shock technique used to assess Ca^2+^-dependent acceleration of recruitment. Reproduction of Figure 2A of Stevens and Wesseling (1998). The RRP was emptied with a pair of osmotic shocks induced with hypertonic solution. Action potentials were evoked during the end of the first osmotic shock at left, but not at right.

Because of these concerns and concerns that Ca^2+^ channel inactivation might additionally complicate measurements of recovery, we began our studies by measuring RRP replenishment with pairs of strong osmotic shocks in the presence and absence of residual Ca^2+^ (Stevens and Wesseling, 1998; see Figure 4).

We accomplished this by exploiting unique features of isolated neurons grown in cell culture on small islands of substrate. The axons of the isolated neurons form synapses onto the dendrites; these are termed *autapses* for auto synapses (Furshpan et al., 1976; Bekkers and Stevens, 1991). The islands can be superfused rapidly with hypertonic solution, allowing osmotic shocks to be induced rapidly (see image in Figure 2). The osmotic shocks drive exocytosis without increasing intracellular Ca^2+^ (Rosenmund and Stevens, 1996), allowing one to follow the time course of recovery by measuring the postsynaptic responses to transmitter released by pairs, much like in experiments where transmitter release is instead driven by action potentials (e.g., Figure 1), except in the absence of the presynaptic Ca^2+^ admitted by action potentials (right panel in Figure 4). Conveniently, the presynaptic terminals of the autapses can be flooded with residual Ca^2+^ by evoking trains of action potentials with the same electrode used to measure postsynaptic responses, additionally allowing measurements of recovery in the presence of residual Ca^2+^ at the same synapses. We found that action potentials evoked during the first osmotic shock of pairs increased the size of the response to the second osmotic shock, indicating that recovery during the interval between shocks was faster (left panel in Figure 4). The increase was driven by residual Ca^2+^ because it was blocked by EGTA, and EGTA had no impact in the absence of action potentials/residual Ca^2+^. A control showing that the action potentials do not alter the sizes of individual quantal events ruled out postsynaptic mechanisms.

## 4. Responses to Osmotic Shocks Reliably Report RRP Replenishment

The controls for confirming that osmotic shocks drive transmitter release from the RRP rather than some other source include co-depletion experiments where trains of action potentials largely eliminate the response to strong osmotic shocks and vice versa (Rosenmund and Stevens, 1996; Moulder and Mennerick, 2005; Garcia-Perez et al., 2008). However, although osmotic shocks do not admit Ca^2+^, they do transiently inhibit Ca^2+^ influx through voltage gated Ca^2+^ channels, which can complicate the interpretation of experiments where osmotic shocks precede trains of action potentials within ~ 10 s (Rosenmund and Stevens, 1996; Garcia-Perez and Wesseling, 2008). Nevertheless, the amount of neurotransmitter released by strong osmotic shocks seems to be equivalent to the amount released during trains of action potentials that exhaust the RRP (Stevens and Williams, 2007), suggesting that strong osmotic shocks can completely exhaust the RRP.

Although the mechanism by which osmotic shocks drive transmitter release is not known, we suspect that physical torsion of the active zone plays a role. Because of this, we were initially concerned that the first shock of each pair might damage the active zone in a way that would alter the timing of vesicle recruitment. However, the pairs of shocks could be repeated many times in individual preparations with reproducible results (Stevens and Wesseling, 1998). And, the time course of recovery was the same in analogous experiments where the hypertonic solution was replaced with hyperkalemic solution or trains of action potentials that were sufficiently intense to nearly completely exhaust the RRP (Stevens and Tsujimoto, 1995; Wesseling and Lo, 2002); hyperkalemic solutions and trains of action potentials generate residual Ca^2+^, so the relevant comparison is to the osmotic shock trials where residual Ca^2+^ was present.

Altogether, these studies provide compelling evidence that osmotic shocks can be used to reliably measure the time course of RRP replenishment, and indicate that residual Ca^2+^ truly does accelerate the mechanism underlying vesicle recruitment. The caveats described above are avoided because the osmotic shocks likely exhaust the RRP. Even if not, we reasoned that the residual Ca^2+^-dependent increase in fusogenicity that is key for the first caveat could not explain the increased amount of release elicited by the second osmotic shocks of pairs because, otherwise, Ca^2+^ would have additionally increased the amount released during single osmotic shocks initiated when the RRP was full, but this was not seen (Stevens and Wesseling, 1999a; e.g., Figure 2). And, the second caveat is avoided because EGTA did not affect the time course of recovery in the absence of residual Ca^2+^.

Note that the logic for ruling out the first caveat is based on information from experiments where the shocks were induced with rapid, strong hypertonic challenges. The caveat might be problematic for weaker hypertonic challenges that do not exhaust the RRP because residual Ca^2+^ would then be expected to increase the aggregate response even when the RRP is full (Rosenmund and Stevens, 1996; Schotten et al., 2015).

In addition, at least one group has reported evidence for basal intracellular Ca^2+^ levels in cultured neurons that were high enough to accelerate the time course of RRP replenishment without additional stimulation (Liu et al., 2014). The result seems to be at odds with our finding that EGTA had no impact on recruitment or fusogenicity in the absence of action potentials. The neurons in Liu et al. (2014) were not autapses, and the presynaptic axon terminals were not voltage clamped by a second electrode, but there were other methodological differences and the ultimate cause of the discrepancy is not known.

## 5. Relevance of Autaptic Synapses to Native Synapses in Tissue

We have not attempted to repeat the osmotic shock experiments at synapses in tissue because of technical difficulties applying and clearing hypertonic solutions rapidly. However, the timing of rate-limiting mechanisms in recruitment to the RRP at autapses—which we grow from dissociated hippocampus—are remarkably similar to at Schaffer collateral synapses in *ex vivo* hippocampal slices. Evidence that recruitment mechanisms are similar in autapses and tissue includes: (1) the time course of RRP replenishment measured with action potentials that matched recovery measured with osmotic shocks above was extracted from experiments at Schaffer collateral synapses; (2) both the use-dependence of induction of supply-rate depression during extended trains of action potentials, and the timing of recovery during subsequent rest intervals, were similar or identical in the two preparations (Stevens and Wesseling, 1999b; Garcia-Perez et al., 2008); (3) the induction of supply-rate depression was selectively accelerated in synapsin knockouts by the same amount in the two preparations (Gabriel et al., 2011); and, finally, (4) the fast rebound in synaptic strength attributed to the transient residual Ca^2+^-dependent enhancement of fusogenicity that is key for the first caveat was initially characterized in autapses and was later found to be intact at Schaffer collateral synapses (Stevens and Wesseling, 1999a; Garcia-Perez and Wesseling, 2008). Taken together, these results suggest that the autapse preparation is a good model for synapses in *ex vivo* tissue, and by extension, *in vivo*, at least for studying rate-limiting mechanisms involved in synaptic vesicle recruitment to the RRP.

In contrast, we do routinely observe striking differences between autapses and Schaffer collateral synapses in phenomena that are downstream of recruitment, such as more paired-pulse depression and more asynchronous release. Even so, the differences do not necessarily lessen the utility of autapses for investigating principles underlying the downstream events because both phenomena are heavily influenced by subtle changes in experimental conditions such as temperature and/or extracellular Ca^2+^ levels, suggesting that the differences are modulatory rather than at the level of basic mechanism. And indeed, there is substantial heterogeneity among Schaffer collateral synapses (e.g., Dobrunz and Stevens, 1997), and some individuals express as much paired-pulse depression as typical autapses. (We have never attempted to measure the extent of variation in asynchronous release among individual Schaffer collateral synapses).

## 6. Controls for Confirming RRP Exhaustion During Trains of Action Potentials

We are not aware of doubts about the conclusion that Ca^2+^ accelerates recruitment to the RRP as a whole beyond the caveats raised in Garcia-Perez and Wesseling (2008), and Ritzau-Jost et al. (2018). However, there are substantial quantitative discrepancies between our estimates of the timing of RRP replenishment during rest intervals and estimates from other groups. We have confidence in our own estimates because, to our knowledge, the discrepancies can always be traced back to experimental designs where time courses are estimated indirectly from the responses to single action potentials or short trains that do not fully exhaust the RRP, which can overestimate the true speed of vesicle recruitment by a large amount owing to the caveats described above. In contrast, mechanisms that are not related to vesicle recruitment should no longer interfere when recovery is extrapolated from the aggregate response to pairs of stimuli that both fully exhaust the RRP.

It is therefore sometimes critical to verify that a candidate frequency of action potentials is sufficient to exhaust the RRP. However, we have found that experiments demonstrating only that synaptic strength depresses to a low steady state level do not guarantee exhaustion, especially of the slow-releasing subdivision (e.g., Figure 3 of Mahfooz et al., 2016). Instead, we verify RRP exhaustion by inducing abrupt jumps to a higher frequency, and include trials where the extracellular Ca^2+^ level is elevated (Wesseling and Lo, 2002; Garcia-Perez and Wesseling, 2008; Mahfooz et al., 2016). In both cases, the absence of an increase in the amount of release is interpreted as evidence that the RRP is exhausted. For the frequency jump experiments, we verify that the axons can follow at the higher frequencies with matched controls after preventing RRP exhaustion by lowering the extracellular Ca^2+^.

Note that the frequency of stimulation required for exhausting the RRP depends very much on the type of synapse, and factors that influence probability of release, such as extracellular Ca^2+^, and must be determined on a case by case basis. For example, 20 Hz was sufficient at Schaffer collateral synapses (Garcia-Perez and Wesseling, 2008), but even 100 Hz was not enough at calyces of Held (Mahfooz et al., 2016).

## 7. Resolving Limits With Trains of Action Potentials

Although the experiments referenced above showed that vesicle recruitment to the RRP is accelerated by Ca^2+^, they did not resolve the extent of the acceleration. That is, in our hands, the osmotic shock technique is limited to rest intervals lasting > 500 ms, and so could have failed to detect acceleration driven by residual Ca^2+^ that was cleared from presynaptic terminals more quickly. This was a real concern in 1998 because residual Ca^2+^ was thought to be cleared in hundreds of ms (Wu and Saggau, 1994).

To address this, we analyzed the rate of transmitter release at times when the RRP was maintained in a near-empty steady state by ongoing stimulation with action potentials. We reasoned that ongoing transmitter release while the RRP is maintained in such a state would necessarily equal recruitment because, otherwise, the RRP would replenish during ongoing stimulation. We could then calculate the minuscule fraction of the RRP that was replenished during the short intervals between action potentials simply by dividing the average amount of release after individual action potentials by the size of the RRP when completely full. The result could be extrapolated to predict the full time course of RRP replenishment during long rest intervals, and the prediction could be compared to actual measurements. A mismatch between prediction and measurement would suggest that a component of the Ca^2+^-dependent acceleration dissipates too quickly to influence the time course of RRP replenishment during subsequent periods of rest.

We did not find any such mismatch for Schaffer collateral synapses beyond the amount already expected from the osmotic shock experiments, indicating that the osmotic shock technique is a reliable tool for measuring the full extent of Ca^2+^-dependent acceleration of vesicle recruitment to the RRP, at least at hippocampal synapses (Wesseling and Lo, 2002). And, later experiments showed that residual Ca^2+^ is cleared in multiple phases, with a slow component taking tens of seconds in Schaffer collaterals that may be the component that modulates the timing of vesicle recruitment (Brager et al., 2003; Garcia-Perez and Wesseling, 2008).

## 8. 10-fold Mismatch at Calyx of Held

We obtained a strikingly different result at calyces of Held, where we found a > 10-fold mismatch between prediction and measurement (Mahfooz et al., 2016). We interpret the result as indicating that the effect of activity on the recruitment mechanism reverses much more quickly at calyces of Held. If so, the widely reported observation that RRP replenishment follows a double exponential function after depleting the RRP a single time at calyces of Held does not necessarily indicate that some RRP subdivisions are replenished more quickly than others. Instead, the time course of replenishment is almost always predicted to be closely approximated by a double exponential function when the timing of recruitment quickly decelerates during rest intervals, even when all subdivisions are replenished at the same rate. The mathematical equation for this is:

(1) $$\begin{array}{r} RRP\left(t\right)=1-e^{-\int \alpha \left(t\right)} \end{array}$$

where *RRP*(*t*) is RRP fullness, and α(*t*) is the unitary rate of recruitment to all subdivisions over time (Wesseling and Lo, 2002; Hosoi et al., 2007; Mahfooz et al., 2016); a unitary rate plays the same role as a rate constant or rate coefficient in standard chemical kinetics, with the difference being that the value of a unitary rate can vary over time.

The conclusion that the effect of activity reverses quickly at calyces of Held is consistent with a role for residual Ca^2+^, which is thought to be cleared much more quickly at calyces of Held compared to Schaffer collateral synapses (Hosoi et al., 2007; Garcia-Perez and Wesseling, 2008). However, unlike for hippocampal synapses, we have not yet devised a method for confirming that Ca^2+^ is indeed the intermediary. Intriguingly, conceptually similar experiments at ribbon synapses within cone photoreceptors in the retina suggest a similarly large mismatch between the timing of recruitment during ongoing stimulation and during subsequent rest intervals (Thoreson et al., 2016).

Notably, the double exponential time course that is characteristic of RRP replenishment after the induction of supply-rate depression at hippocampal synapses is caused by a completely different type of mechanism likely involving depletion of reserve stores, and does not involve Ca^2+^ (Stevens and Wesseling, 1999b; Gabriel et al., 2011). Supply-rate depression can be easily isolated from residual Ca^2+^-dependent mechanisms because it is not induced until after exocytosis of the equivalent contents of several RRPs, which takes > 6 s at room temperature when the frequency of stimulation is maximal (Garcia-Perez et al., 2008).

## 9. Caveats Related to Estimating RRP Capacity

A variety of procedures have been employed for extracting information about RRP size from synaptic responses evoked by trains of presynaptic action potentials (Wesseling and Lo, 2002; Neher, 2015; Thanawala and Regehr, 2016; Thoreson et al., 2016). In our case, correct estimates were critical for estimating the timing of recruitment during ongoing stimulation. We avoided procedures that are based on the premise that all readily releasable vesicles undergo exocytosis with the same probability following individual action potentials because the premise is not compatible with the concept that RRPs contain both fast- and slow-releasing subdivisions, and can lead to underestimates of the true size when the slow subdivision is large. This was important for our analysis because such methods would have produced order of magnitude sized overestimates of the timing of vesicle recruitment in some cases, at least for calyces of Held where the RRPs in some preparations contained slow-releasing subdivisions that were almost 10-fold greater than the fast-releasing subdivision (Figure 9 of Mahfooz et al., 2016).

We avoided the premise by first summing up the amount of release during trains of stimulation that fully exhaust the RRP, and then subtracting the fraction of the sum generated by transmitter that was newly recruited during stimulation (Wesseling and Lo, 2002; Mahfooz et al., 2016). The procedure does not depend on assumptions about the distribution of release probabilities among readily releasable vesicles, but does depend on assumptions about mechanisms underlying recruitment that are not yet fully resolved. An evaluation of the full range of possibilities, however, indicated that even extreme assumptions do not alter the estimates by more than 25 % (Figure 7 of Mahfooz et al., 2016), which is not enough to alter the conclusion that recruitment at the calyx of Held is much faster during ongoing stimulation than during subsequent periods of rest.

Even the small range of uncertainty in estimates of RRP size could be largely eliminated by incorporating the premise that RRPs have a fixed capacity for storing vesicles (Mahfooz et al., 2016). The premise is supported by: (1) evidence that intracellular Ca^2+^ does not alter the number of readily releasable vesicles at resting synapses, when the RRP would be full, despite accelerating recruitment of new vesicles when partially empty (Stevens and Wesseling, 1999a); and, (2) a mathematical analysis that found straightforward relationships between the results of frequency jump experiments where the RRP was first driven to a variety of partially full steady state levels using submaximal frequencies for stimulation followed by abrupt jumps to a higher frequency that then exhausted the RRP (Mahfooz et al., 2016; Raja et al., 2019). Experiments demonstrating that RRP size at resting synapses was not altered when synaptic strength was increased 2-fold by increasing extracellular Ca^2+^ ruled out specific alternatives that have been proposed (Wesseling and Lo, 2002; Mahfooz et al., 2016). Thoreson et al. (2016) concluded that RRPs also have a fixed capacity at ribbon type synapses in cone photoreceptors using a largely independent line of reasoning.

The premise of a fixed capacity is particularly relevant because it matches the physical interpretation that the RRP is made up of vesicles that are docked to a stable collection of release sites (e.g., Figure 3).

## 10. Paradigm Level Concerns

The evidence for a fixed capacity is additionally relevant because it addresses concerns about the utility of the very concept of an RRP for understanding the physiology of synaptic transmission (see Pan and Zucker, 2009). For example, endocytosis of spent vesicular membrane occurs by a variety of modes, the fastest of which is termed *kiss-and-run* exo/endocytosis, and may occur in milliseconds (Alabi and Tsien, 2013). At least one study has concluded that vesicles undergoing kiss-and-run can be fully recycled more rapidly than recruitment to the RRP (Pyle et al., 2000). If so, the rapidly recycled vesicles could, in principle, contribute multiple quanta of neurotransmitter to estimates of RRP size even though only one of the quanta would be truly readily-releasable at any given time. However, such a mechanism does not seem to be consistent with the evidence that RRP capacity is not affected by extracellular or intracellular Ca^2+^ because kiss-and-run is modulated by both (Harata et al., 2006; Richards, 2010; Leitz and Kavalali, 2011). Additional observations arguing against a role for kiss-and-run in estimates of RRP size at Schaffer collateral synapses and calyces of Held include evidence that: (1) RRP size is not influenced by the time taken to exhaust the RRP over a large range, extending from 30–1000 ms at calyces of Held (Chen et al., 2015; Mahfooz et al., 2016), whereas very fast recycling would be expected to have more of an impact in experiments where the time taken is longer; (2) the amount of kiss-and-run is less at synapses with higher probability of release, but increasing the probability by deleting synaptophysin family proteins did not alter estimates of RRP capacity (Raja et al., 2019; see Gordon et al., 2011 and Kwon and Chapman, 2011 for evidence that endocytosis is slower in synaptophysin knockouts); and, (3) RRP capacity is consistently similar or less, than the number of docked vesicles measured with morphological techniques (Schikorski and Stevens, 2001; Neher, 2017; see von Gersdorff et al., 1996 for a different result at ribbon synapses). In any case, the maximum speed of vesicle recycling after kiss-and-run continues to be debated, but most estimates seem to be slower or equivalent to recruitment to the RRP (Aravanis et al., 2003; Richards, 2010; Alabi and Tsien, 2013).

## 11. Molecular Effectors

We have not made much progress toward identifying effectors of residual Ca^2+^ that accelerate vesicle recruitment to the RRP. Part of the difficulty is that the rate-limiting mechanism in recruitment has not yet been identified. Still viable possibilities include: locomotion of vesicles to docking sites in the active zone; a post-docking priming step; or even post-exocytosis reconstitution of release sites (Neher, 2010). At present, it seems that kiss-and-run exo/endocytosis is probably not involved, however, because Ca^2+^ is thought to block rather than accelerate this phenomenon (Harata et al., 2006; Richards, 2010; Leitz and Kavalali, 2011). In any case, we can at least rule out our initial hypothesis that synapsin proteins are involved (Gabriel et al., 2011; see also Gaffield and Betz, 2007).

Liu et al. (2014) reported a Ca^2+^-dependent component of basal recruitment that was absent in synaptotagmin 7 knockout synapses. The result suggests that synaptotagmin 7 may be involved, but a wide range of other functions for synaptotagmin 7 have been reported by other groups (Bacaj et al., 2013; Jackman et al., 2016; Chen et al., 2017). Notably, although Liu et al. (2014) did use osmotic shocks to monitor RRP replenishment, the experimental design differed from our own in key regards including: enough basal Ca^2+^ to activate the Ca^2+^-dependent component of recruitment; and hypertonic challenges lasting 10 s, vs. only 3 or 4 in our case, suggesting that the shocks were weaker.

Other groups have concluded that Ca^2+^ accelerates vesicle trafficking at calyces of Held via calmodulin and Munc 13-1 (reviewed in Ritzau-Jost et al., 2018). However, our understanding has been that the calmodulin pathway is thought to accelerate transfer of vesicles from the slow- to fast-releasing subdivision of the RRP, which would be downstream of vesicle recruitment to the RRP as a whole, and therefore a different category of mechanism (Sakaba and Neher, 2001; Hosoi et al., 2007; but see Van Hook et al., 2014); some other explanation will be required for key results if it turns out that vesicles are recruited to slow- and fast-releasing subdivisions in parallel. In any case, it seems that the Munc 13-1^W464R^ mutation thought to disrupt the calmodulin pathway did not eliminate the mismatch between the timing of vesicle recruitment during ongoing stimulation and during subsequent rest intervals (Lipstein et al., 2013). The steady state response at mutant calyces of Held was not altered (their Figure 7C), but recovery during rest intervals was slower (their Figure 5D3), suggesting that the mutation actually made the mismatch greater. These results support the idea that calmodulin acts downstream of recruitment to the RRP, at least at calyces of Held. If so, the mechanism could have an important impact during light or moderate use that might be superseded as rate-limiting during heavy use by the upstream step whereby vesicles are initially recruited to the RRP as a whole. Myosine light chain kinase has also been implicated in regulating short-term plasticity during light and moderate use without affecting release as much during heavy use (Srinivasan et al., 2008; González-Forero et al., 2012).

## 12. Summary

Multiple concerns have been raised that could complicate the interpretation of some experiments designed to measure activity and residual Ca^2+^-dependent acceleration of the mechanism by which vesicles are recruited to the RRP. However, we continue to be confident that residual Ca^2+^ does accelerate the recruitment mechanism, at least at hippocampal synapses, because our own experiments in this area were designed to avoid the underlying caveats. A key methodological point is that multiple aspects of our experimental design depended critically on stimulation protocols that are sufficiently intense to nearly completely exhaust both fast- and slow-releasing subdivisions of the RRP, and that control experiments designed to confirm that both are truly exhausted need to be more sophisticated than simply observing that stimulation drives neurotransmitter release to a low steady state. A second point is that the calmodulin/Munc 13 pathway implicated in modulating vesicle trafficking seems to operate downstream of vesicle recruitment to the RRP, and that the molecules responsible for accelerating recruitment at the upstream step whereby vesicles are recruited to the RRP as a whole remain to be determined. The list of presynaptic protein families with no known function remains long, so there is no shortage of candidates.

## Author Contributions

The author confirms being the sole contributor of this work and has approved it for publication.

### Conflict of Interest

The author declares that the research was conducted in the absence of any commercial or financial relationships that could be construed as a potential conflict of interest.
